# Physical Features and Vital Signs Predict Serum Albumin and Globulin Concentrations Using Machine Learning

**DOI:** 10.31557/APJCP.2021.22.2.333

**Published:** 2021-02

**Authors:** Jing Wei, Jie Xiang, Yousef Yasin, Andrew Barszczyk, Deanne Tak On Wah, Meifen Yu, Wendy Wenyu Huang, Zhong-Ping Feng, Kang Lee, Hong Luo

**Affiliations:** 1 *The Affiliated Hospital of Hangzhou Normal University, Hangzhou Normal University. Hangzhou, Zhejiang, People’s Republic of China. *; 2 *Department of Applied Psychology and Human Development, Ontario Institute for Studies in Education, University of Toronto, Toronto, Ontario, Canada.*; 3 *Department of Physiology, University of Toronto, Toronto, Ontario, Canada. *

**Keywords:** Anthropometry, blood pressure, pulse, health screening, blood biomarker prediction

## Abstract

**Objective:**

Serum protein concentrations are diagnostically and prognostically valuable in cancer and other diseases, but their measurement via blood test is uncomfortable, inconvenient, and costly. This study investigates the possibility of predicting albumin, globulin, and albumin-globulin ratio from easily accessible physical characteristics (height, weight, Body Mass Index, age, gender) and vital signs (systolic blood pressure, diastolic blood pressure, mean arterial pressure, pulse pressure, pulse) using advanced machine learning techniques.

**Methods:**

We obtained albumin concentration, globulin concentration, albumin-globulin ratio and predictor information (physical characteristics, vital signs) from physical exam records of 46,951 healthy adult participants in Hangzhou, China. We trained a computational model to predict each serum protein concentration from the predictors and then evaluated the predictive accuracy of each model on an independent portion of the dataset that was not used in model training. We also determined the relative importance of each feature within the model.

**Results:**

Prediction accuracies were r=0.540 (95% CI: 0.539-0.540; Pearson r) for albumin, r=0.250 (95% CI: 0.249-0.251) for globulin, and r=0.373 (95% CI: 0.372-0.374) for albumin-globulin ratio. The most important predictive features were age (100% ± 0.0%; mean ± 95% CI of normalized importance), gender (34.4% ± 0.7%), pulse (25.6% ± 1.3%) and Body Mass Index (24.4% ± 2.3%) for albumin, pulse (83.7% ± 3.8%) for globulin, and age (99.2% ± 1.0%), gender (59.2% ± 1.7%), Body Mass Index (46.1% ± 4.2%) and height (40.0% ± 3.8%) for albumin-globulin ratio.

**Conclusions:**

Our models predicted serum protein concentrations with appreciable accuracy showing the promise of this approach. Such models could serve to augment existing tools for identifying “at-risk” individuals for follow-up with a blood test.

## Introduction

Clinical blood panels that measure albumin and globulin proteins in blood serum are valuable diagnostic and prognostic tools. Albumins function to maintain osmotic pressure (Busher, 1990) and transport materials throughout the bloodstream (He et al., 2017a). Low albumin levels are a symptom of cancers, as well as liver disease, kidney disease, inflammation, and malnutrition; they are further associated with increased risk of general and cardiovascular mortality (Goldwasser and Feldman, 1997). Globulins function as enzymes, carrier proteins, and immune cells (Busher, 1990). Similar to albumins, low globulin levels are a symptom of liver disease, kidney disease, and malnutrition (Teloh, 1978; Busher, 1990). High globulin levels are a symptom of chronic inflammation, acute infections, and some cancers including multiple myeloma and lymphoma (Busher, 1990). Albumin-globulin ratio (AGR), which is calculated as albumin / (total protein – albumin), is considered to be a strong prognostic tool for many cancers, as it combines the effectiveness of both albumin and globulin in predicting survival outcomes, particularly where solid tumors are involved (He et al., 2017a). Despite the immense relevance of these biomarkers to cancers and other diseases, barriers to blood testing (e.g., expense, discomfort, inconvenience) limit their routine use in most people. Homeostatic imbalances therefore go undetected until diseases progress to more advanced stages. 

This problem could be addressed by creating computational models that predict these serum protein concentrations from easily accessible patient characteristics (e.g., physical features and vital signs). Such data-driven tools are becoming possible with the recent proliferation of large clinical datasets of electronic health records (‘Big Data’); such datasets have several advantages. First, they encompass a clinically diverse sample of the overall population rather than disease-specific patient groups typical of clinical studies (Obermeyer and Emanuel, 2016). Findings should therefore generalize well to all patients. Second, such datasets collect a plethora of variables without prior specific hypotheses for their use. This allows hypotheses to be generated and tested after data collection has taken place, and it facilitates the identification of new variables for explaining complex biological systems (hypothesis-generating research) (Hulsen et al., 2019). Third, large datasets provide the requisite statistical power for identifying subtle relation between variables. 

Physical features and vital signs have known associations with albumin, globulin, and AGR. In terms of physical characteristics, albumin levels decrease and globulin levels increase with age, and so AGR decreases as individuals age (Bender et al., 1975; Eustace et al., 2004; Montazerghaem et al., 2014). Furthermore, Body Mass Index (BMI) and AGR are positively correlated. This relation holds true in tuberculosis and cancer patients with low BMI, as they also have lower AGR (Sultan et al., 2012; Zhou et al., 2016). In terms of vital signs, there is only limited research on the associations between serum albumins or globulins with measures of cardiac health such as heart rate and blood pressure. There seems to be no relation between heart rate and serum albumin levels (Uthamalingam et al., 2010). However, serum albumin levels are elevated in patients with high blood pressure (Salako et al., 2003; Høstmark et al., 2005; Uthamalingam et al., 2010), even though albumin is negatively associated with cardiovascular disease. It has been suggested that albumin and blood pressure affect cardiovascular health through different and unrelated mechanisms (Høstmark et al., 2005). Little is known about the association of globulins with heart rate and blood pressure, and further research is required in this area. 

Modeling the relation between predictive features and serum proteins will require advanced machine learning techniques; such techniques have become practical given recent advances in computational power. Advanced machine learning algorithms (e.g., multilayer perceptron) can model complex multi-dimensional and non-linear relationships between variables; this is not easily accomplished with traditional statistical techniques like regression (Mullainathan and Spiess, 2017). Machine learning algorithms model such relationships automatically and efficiently, even for highly complex models with numerous predictors (Mullainathan and Spiess, 2017). Such algorithms are therefore ideal for modeling yet-unknown interactions among variables in complex systems,as in the case of combining the information contained within physical features and vital signs to predict serum protein concentrations. In this way, big data and advanced machine learning techniques could enable the creation of tools that predict blood contents with reasonable accuracy. Such tools could be used to recommend ‘at-risk’ patients for follow-up or more frequent blood testing.

This study will investigate the possibility of predicting albumin concentration, globulin concentration, and albumin-globulin ratio in blood serum from a set of easily accessible patient physical features and vital signs. We will model these relationships in a general population of Chinese adults. We will do so using advanced machine learning techniques to facilitate efficient combination of features. We will then evaluate the predictive accuracy of each model on a portion of the dataset that was not used in creating these models. We anticipate that albumin, globulin and albumin-globulin ratio can be predicted with reasonable accuracy from physical characteristics (height, weight, Body Mass Index, age, gender) and vital signs (systolic blood pressure, diastolic blood pressure, mean arterial pressure, pulse pressure, pulse) using machine learning. We will evaluate our findings in the context of correlations between predictive features and serum protein concentrations identified in other studies. This work will help identify practical and accessible features for inferring serum protein concentrations, which in turn will help build diagnostic and prognostic tools for identifying ‘at-risk’ individuals who are likely to benefit from further blood testing. 

## Materials and Methods

*Participants*

The present study included 46,951 healthy adult participants (mean age=40; SD=13.9) as they completed their routine physical examination at the Health Management Centre at the Affiliated Hospital of Hangzhou Normal University. Participants included 42.8% males and 57.2% females. The study was conducted in accordance with NIH research ethics guidelines and was approved by Research Ethics Review Committee at the Affiliated Hospital of Hangzhou Normal University. Participants provided written informed consent for the use of their data in this study. 

*Data Collection Procedure*

Participants were seen individually by medical practitioners. Blood samples were collected using needles and sample tubes. Demographic information including age was recorded, height and weight was measured, and pulse and blood pressure assessed. 

*Calculations*

Age was calculated in years, from the date of birth to the date of examination. Height was measured using a stadiometer and recorded in meters. Weight was measured using a weighing scale and recorded in kilograms. Measures of height and weight were then used to calculate Body Mass Index (Equation 1).

$\mathrm{Body Mass Index}\left(\mathrm{BMI}\right)=\mathrm{Weight}\frac{\mathrm{kg}}{\mathrm{Height}}{\left(m\right)}^{2}$

*Equation 1*

Pulse (beats-per-minute) was measured using a stethoscope. Blood pressure was measured using a stethoscope and sphygmomanometer. Measures of blood pressure were then used to calculate mean arterial pressure (Equation 2). 

Mean Arterial Presure MAP=Systolic Blood Presure+2(Diastolic Blood Presure)3

*Equation 2*

Pulse pressure was calculated as the difference between systolic and diastolic pressures.

*Blood Analysis*

Blood samples were sent for total serum protein Test, which measures the amount of protein in each blood sample including albumin and globulin. This then allowed for calculations of albumin-globulin ratio. 

*Data Analysis*

Outliers that were 3 standard deviations above or below the mean were removed from the dataset. Three independent prediction models were created for albumin, globulin, and AGR using a multilayer perceptron neural network (MLPNN). The inputs of this machine learning algorithm (SPSS, Version 24) were age, Body Mass Index (BMI), pulse, height, weight, gender, pulse pressure, mean arterial pressure (MAP), systolic blood pressure, and diastolic blood pressure. In order to develop the models, the participant sample was randomly divided into a training set (70%), testing set (15%), and validation set (15%). The training and testing sets were used as their name suggests to train and test prediction models. An independent validation set was selected to evaluate the models’ ability to predict serum protein concentrations. The procedures for training, testing, and validating models were repeated for 100 iterations for each serum protein to generate statistical estimates of model performance and normalized feature importance.

Model performance was calculated as explained variance (R^2^) on the validation set. An overall Pearson correlation (r) was calculated by taking the square root of the mean explained variance and its 95% confidence interval across all 100 iterations of each model. In each iteration, the feature importance function of SPSS was used to determine the importance of each feature. This value was normalized against the best-performing feature to obtain a relative importance (%) for each feature. The mean normalized importance and its 95% confidence interval was calculated across all 100 iterations for each model. A scatterplot was created to plot observed versus predicted albumin, globulin, and AGR using in the 100th iteration of each model. 

## Results

**Figure 1:**
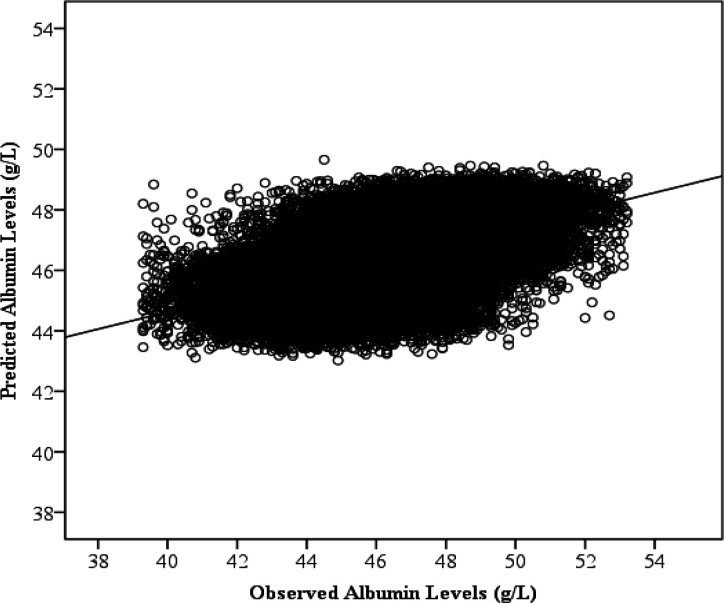
Observed and Predicted Albumin Levels (g/L). Each point represents a participant’s albumin level and the predicted albumin level using age, Body Mass Index, pulse, height, weight, gender, pulse pressure, mean arterial pressure, systolic blood pressure, and diastolic blood pressure as predictors. A line of best fit is drawn through the points to illustrate the significant positive correlation between the observed and predicted globulin levels using a multilayer perceptron neural network. This plot depicts predictions from the 100^th^ iteration of the model

**Figure 2:**
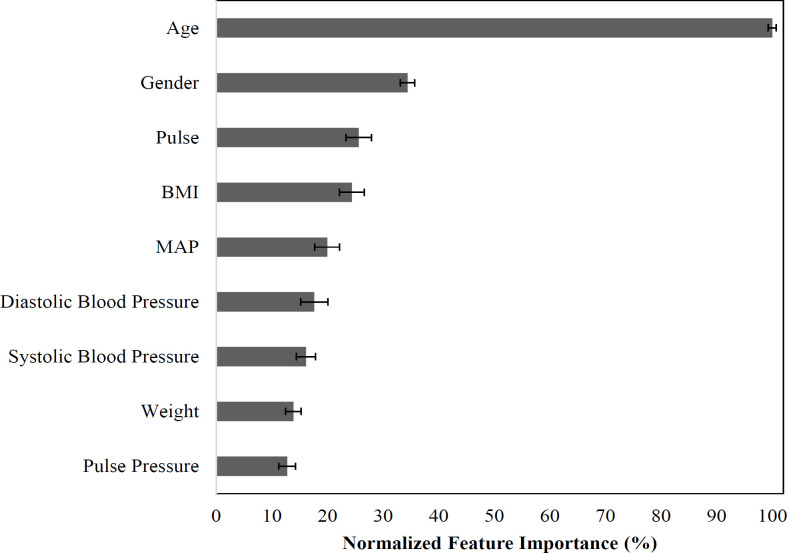
Albumin Model Feature Importance. For each of 100 model iterations, feature importance was calculated and then normalized to the most important feature and expressed as a percentage. Values are the mean normalized feature importance and its 95% confidence interval across 100 model iterations. BMI, Body Mass Index; MAP, Mean Arterial Pressure

Our study tested the hypothesis that the physical features and vital signs of age, BMI, gender, systolic blood pressure, diastolic blood pressure, pulse, height, weight, MAP, and pulse pressure predict albumin concentration, globulin concentration and AGR using MLPNN. The scatterplot in Figure 1 shows the observed versus predicted albumin levels (g/L) using MLPNN prediction models. The regression line shows the correlation between the observed and predicted results (r=.540; 95% CI: 0.539-0.540; Pearson r). Importance for the variables in predicting albumin levels are shown in Figure 2. Across the 100 iterations, age is by far the most important (contains the most information about albumin concentration) (mean ± 95% CI: 100% ± 0.0%), followed by gender (34.4% ± 0.7%), pulse (25.6% ± 1.3%) and BMI (24.4% ± 2.3%), which were less than half as important.

**Figure 3:**
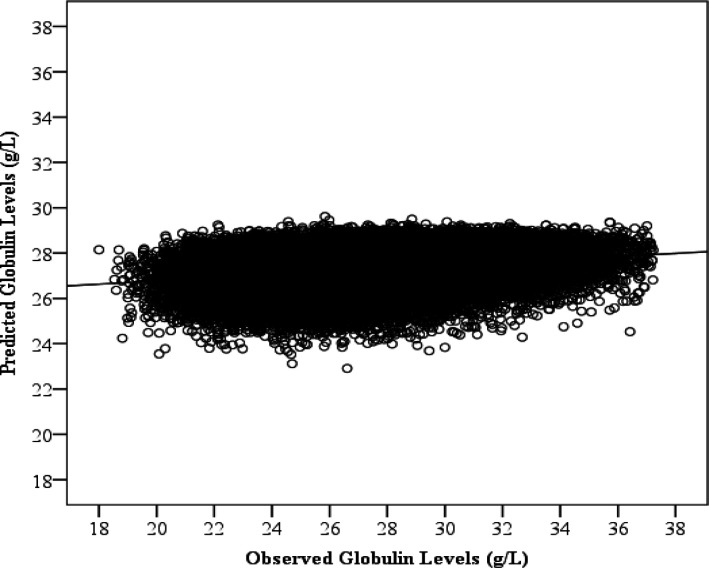
Observed and Predicted Globulin Levels (g/L). Each point represents a participant’s globulin levels and predicted globulin levels using age, Body Mass Index, pulse, height, weight, gender, pulse pressure, mean arterial pressure, systolic blood pressure, and diastolic blood pressure as predictors. A line of best fit is drawn through the points to illustrate the significant positive correlation between the observed and predicted globulin levels using a multilayer perceptron neural network. This plot depicts predictions from the 100^th^ iteration of the model

**Figure 4:**
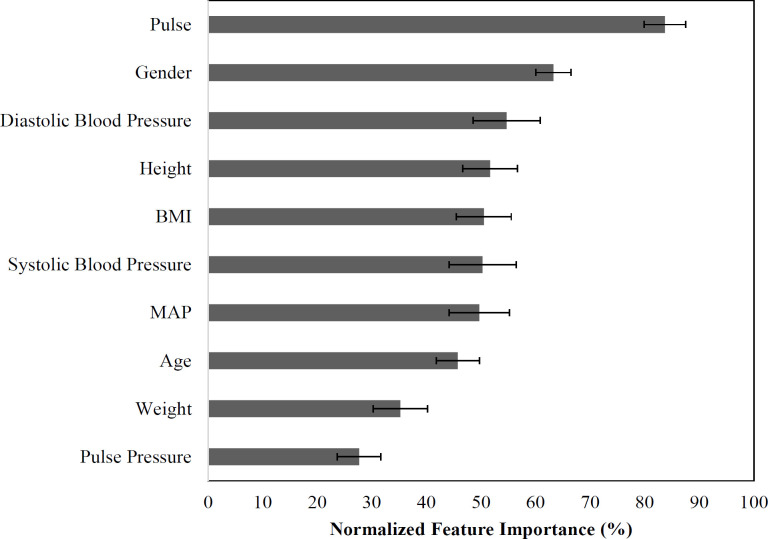
Globulin Model Feature Importance. For each of 100 model iterations, feature importance was calculated and then normalized to the most important feature and expressed as a percentage. Values are the mean normalized feature importance and its 95% confidence interval across 100 model iterations. BMI, Body Mass Index; MAP, Mean Arterial Pressure

The scatterplot in Figure 3 shows the observed versus predicted globulin levels (g/L) using MLPNN prediction models. The regression line shows the correlation between the observed and predicted globulin levels (r=0.250; 95% CI: 0.249-0.251). Contrary to the previous models, the most important features for predicting globulin was pulse (a physiological feature) which accounts for the most information (83.7% ± 3.8%). Gender (63.3% ± 3.2%), diastolic blood pressure (54.7% ± 6.1%), height (51.7% ± 5.1%), BMI (50.5% ± 5.0%), systolic blood pressure (50.3% ± 6.1%), MAP (49.7% ± 5.5%), age (45.8% ± 4.0%), and weight (35.4% ± 5.0%) were about half as important (Figure 4).

**Figure 5:**
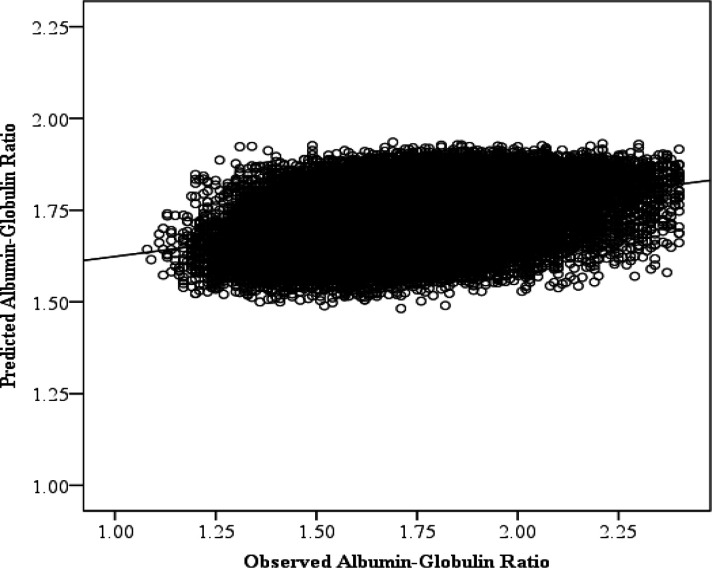
Observed and Predicted Albumin-Globulin Ratio (AGR). Each point represents a participant’s AGR and the predicted AGR using age, Body Mass Index, pulse, height, weight, gender, pulse pressure, mean arterial pressure, systolic blood pressure, and diastolic blood pressure as predictors. A line of best fit is drawn through the points to illustrate the significant positive correlation between the observed and predicted AGR using a multilayer perceptron neural network. This plot depicts predictions from the 100^th^ iteration of the model

**Figure 6:**
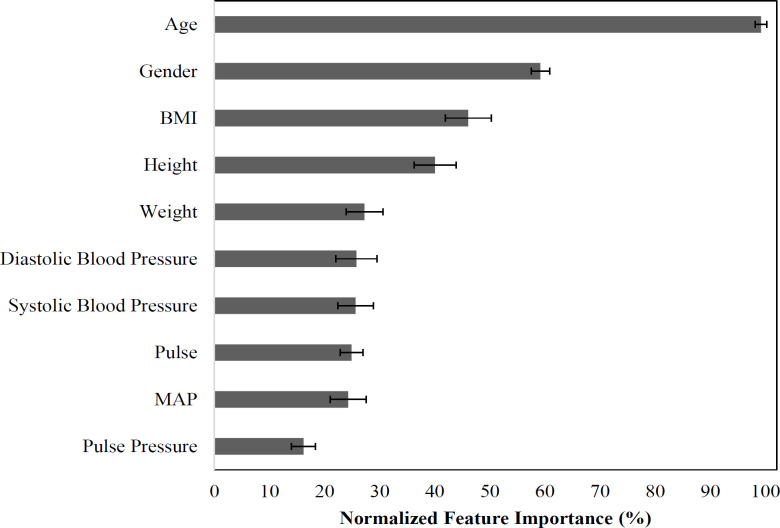
Albumin-Globulin Ratio (AGR) Model Feature Importance. For each of 100 model iterations, feature importance was calculated and then normalized to the most important feature and expressed as a percentage. Values are the mean normalized feature importance and its 95% confidence interval across 100 model iterations. BMI, Body Mass Index; MAP, Mean Arterial Pressure

The scatterplot in Figure 5 shows the observed versus predicted AGR using MLPNN prediction models. The regression line shows the correlation between the predicted and observed AGR (r=.373; 95% CI: 0.372-0.374). Similar to albumin alone, age was once again the most important (99.2% ± 1.0%). Gender (59.2% ± 1.7%) and BMI (46.1% ± 4.2%) were also top features, as is height (40.0% ± 3.8%); they were approximately half as important as age. Unlike with albumin, pulse (24.9% ± 2.1%) is no longer a top feature (Figure 6).

## Discussion

In this study, we created computational models for predicting albumin concentration, globulin concentration, and AGR using the physical features and vital signs of age, BMI, pulse, weight, height, systolic blood pressure, diastolic blood pressure, pulse pressure, and MAP. Based upon the results of the present study, it is reasonable to suggest that albumin and AGR can both be predicted well using these variables. 

We were best able to predict albumin levels in this study. Since albumin is produced in the liver, it is not surprising that physical features and vital signs associated with liver health, namely age, weight, and gender held greater weight in the model. External stressors begin taking a greater toll on the body as people age (Kim et al., 2015), and this is associated with increased risk for major diseases including cancers, cardiovascular disorders, and neurodegenerative diseases (López-Otín et al., 2013). Liver volume and blood flow decrease with age (Wynne et al., 1989; Zoli et al., 1999); hence, production of albumin decreases with age. Furthermore, there is a relation between albumin levels and weight. For instance, albumin concentration is associated with weight loss in cancer patients (McMillan et al., 2001) and serum albumin levels collected at admission to a hospital can predict weight loss in children during their stay (Quadros et al., 2019). Albumin is also a useful indicator of nutritional status. As such, the strong link between weight and albumin is not surprising. Furthermore, it has been suggested that the immune response during illness uses amino acids from available proteins (including albumin) to synthesize acute-phase proteins (McMillan et al., 2001). Hence, serum albumin levels may decrease during illness. The present study found that gender was important for predicting albumin levels. Although there is limited research in Chinese adults, a study conducted in the United Kingdom may explain why gender impacts albumin levels. It appears that females between the ages of 20 and 50 in the United Kingdom tend to have lower serum albumin levels than males; researchers have attributed this to the use of oral contraceptives (Weaving et al., 2016). However, the applicability of this finding to the Chinese females in our study may differ, since pharmaceutical and oral contraceptive use may differ between the two countries.

The model predicting globulin concentration was less successful than models predicting albumin concentration and AGR. Since globulin is comprised of many different proteins including enzymes, carrier proteins, and immunoglobins, its’ roles within the body are much more diverse. We did not have additional indicators (e.g., indicators of immune status) that could help us account for these factors in our work, and so accurately predicting immunoglobin levels was a challenge. There is evidence that in rare cases, immunoglobin therapy can result in arrhythmia, hypotension, and renal impairment (Guo et al., 2018). However, this link is not well understood. Further research may be warranted in this area.

AGR is calculated from albumin and globulin concentrations, and so the predictability of AGR from the same physical features and vital signs should be within the range of predictability as indicated through the albumin and globulin models. Previous research has shown that AGR is a better predictor of mortality than albumin level alone (Azab et al., 2013), so the utility of this measure is high compared to albumin alone. In fact, healthy individuals with low AGR as a result of underproduction of albumin and over production of globulin have a higher risk for major cancer types, especially liver and hematological cancers (Suh et al., 2014). In cancer patients, low AGR predicts poorer survival outcomes (He et al., 2017a) and is associated with older age (Bi et al., 2016; He et al., 2017b; Wang et al., 2019) and lower BMI (Bi et al., 2016; Zhou et al., 2016; He et al., 2017b; Wang et al., 2019). In rare cases, AGR may be high, indicating an underproduction of immunoglobulins typically characteristic of leukemia patients. However, this may also simply reflect dehydration. AGR is further associated with other health conditions such as heart failure, cirrhosis, liver malfunction, nephrotic syndrome, autoimmune diseases, chronic inflammation (Duran et al., 2014), and rheumatoid arthritis (Lefkovits and Farrow, 1955). 

Overall, our research shows that physical features and vital signs are useful predictors albumin concentration, globulin concentration, and AGR, which in turn are useful predictors of health status and outcomes. These predictors can be obtained quickly, easily and non-invasively with readily available tools; such models could therefore be useful tools for inferring health status and prognostic outcomes. ‘At-risk’ patients could then be referred for more in-depth follow-up with a medical professional. 

The present study uniquely employs larger sample size than previously used (He et al., 2017a; Wang et al., 2019), and this has likely contributed to the creation of robust models in the current study. 

Although this research has clear benefits, there are some limitations to the study. Firstly, the participants were recruited from a specific hospital associated with Hangzhou Normal University in China. Further, the majority of participants were ethnically Chinese. To ensure generalizability of the present study beyond this population, it will be important to reproduce these results using participants from other ethnic backgrounds and socio-cultural settings. Future studies could also validate these models in specific disease populations to ensure that they generalize. 

There is significant opportunity to further increase the prediction accuracy of these models. One way is by adding features containing additional information about serum protein concentrations. Novel digital ‘biomarkers’ like continuous physiological information measured by wearables or contactless technologies (Luo et al., 2019) could constitute at least one non-invasive yet robust source of information to investigate in future studies. Prediction accuracy could be further improved by optimizing the machine learning algorithm that is used to create the model. While a specific algorithm type (e.g., neural network, random forest, support vector machine) may be well suited to a certain prediction task (Marsland, 2015), the optimal algorithm for any task (and its ‘hyperparameters’) must be determined empirically. Future studies should determine the algorithm and hyperparameters that maximize model performance. 

*List of Abbreviations*

AGR                  albumin-globulin ratio

BMI                   Body Mass Index

SD                     standard deviation

MAP                  mean arterial pressure

MLPNN             multilayer perceptron neural network

## References

[1] Azab BN, D M, Bhatt VR (2013). Value of the pretreatment albumin to globulin ratio in predicting long-term mortality in breast cancer patients. Am J Surg.

[2] Bender AD, Post A, Meier JP, Higson JE, Reichard G (1975). Plasma protein binding of drugs as a function of age in adult human subjects. J Pharm Sci.

[3] Bi X, Wang L, Zhang W, Yan S (2016). The pretreatment albumin to globulin ratio predicts survival in patients with natural killer/ T-cell lymphoma. Peer J.

[5] Duran AO, Inanc M, Karaca H (2014). Albumin-globulin ratio for prediction of long-term mortality in lung adenocarcinoma patients. Asian Pac J Cancer Prev.

[6] Eustace J, Astor B, Muntner P, Ikizler T, Coresh J (2004). Prevalence of acidosis and inflammation and their association with low serum albumin in chronic kidney disease. Kidney Int.

[7] Goldwasser P, Feldman J (1997). Association of serum albumin and mortality risk. J Clin Epidemiol.

[8] Guo Y, Tian X, Wang X, Xiao Z (2018). Adverse effects of immunoglobulin therapy. Front Immunol.

[9] He J, Pan H, Liang W (2017a). Prognostic Effect of Albumin-to-Globulin Ratio in Patients with solid tumors: A Systematic Review and Meta-analysis. J Cancer.

[10] He X, Guo S, Chen D (2017b). Preoperative albumin to globulin ratio (AGR) as prognostic factor in renal cell carcinoma. J Cancer.

[11] Hong, Deye Y, Andrew B (2019). Smartphone-based blood pressure measurement using Transdermal Optical Imaging technology. Circulation Cardiovascular Imaging.

[12] Høstmark AT, Tomten SE, Berg JE (2005). Serum albumin and blood pressure: a population-based, cross-sectional study. J Hypertension.

[13] Hulsen T, Jamuar SS, Moody AR (2019). From big data to precision medicine. Front Med.

[14] Kim H, Kisseleva T, Brenner DA, Diego S (2015). Aging and liver disease. Curr Opin Gastroenterol.

[15] Lefkovits AM, Farrow IJ (1955). The liver in rheumatoid arthritis. Ann Rheum Dis.

[16] López-Otín C, Blasco MA, Partridge L, Serrano M, Kroemer G (2013). The hallmarks of aging. Cell.

[18] McMillan DC, Watson WS, O’Gorman P (2001). Albumin concentrations are primarily determined by the body cell mass and the systemic inflammatory response in cancer patients with weight loss. Nutr Cancer.

[19] Montazerghaem H, Safaie N, Nezhad VS. (2014). Body mass index or serum albumin levels: Which is further prognostic following cardiac surgery?. J Cardiovasc Thorac Res.

[21] Obermeyer Z, Emanuel EJ (2016). Predicting the future-big data, machine learning, and clinical medicine. N Engl J Med.

[22] Quadros DS, Kamenwa R, Akech S, Macharia W (2019). Pre-albumin as a marker for predicting weight loss in hospitalised children. South Afr J Clin Nutr.

[23] Salako BL, Odukogbe ATA, Olayemi O (2003). Serum albumin, creatinine, uric acid and hypertensive disorders of pregnancy. East Afr Med J.

[24] Suh B, Park S, Shin DW (2014). Low albumin-to-globulin ratio associated with cancer incidence and mortality in generally healthy adults. Ann Oncol.

[25] Sultan KM, Alobaidy MW, AL-Jubouri AM, Naser AA, AL-Sabah HA (2012). Assessment of body mass index and nutritional status in pulmonary tuberculosis patients. J Fac Med Baghdad.

[26] Teloh HA (1978). Serum proteins in hepatic disease. Ann Clin Lab Sci.

[27] Uthamalingam S, Kandala J, Daley M, Januzzi JL (2010). Serum albumin and mortality in acutely decompensated heart failure. Am Heart J.

[28] Wang N, Liu J, Li X (2019). Pretreatment serum albumin/globulin ratio as a prognostic biomarker in metastatic prostate cancer patients treated with maximal androgen blockade. Asian J Androl.

[29] Weaving G, Batstone GF, Jones RG (2016). Age and sex variation in serum albumin concentration: an observational study. Ann Clin Biochem.

[30] Wynne HA, Cope LH, Mutch E (1989). The effect of age upon liver volume and apparently liver blood flow in healthy man. Hepatology.

[31] Zhou T, He X, Fang W, Zhan J, Hong S (2016). Pretreatment albumin/ globulin ratio predicts the prognosis for small-cell lung cancer. Medicine.

[32] Zoli M, Magalotti D, Bianchi G (1999). Total and functional hepatic blood flow decrease in parallel with ageing. Age Ageing.

